# The SNARE Protein Syntaxin 3 Confers Specificity for Polarized Axonal Trafficking in Neurons

**DOI:** 10.1371/journal.pone.0163671

**Published:** 2016-09-23

**Authors:** Linda Soo Hoo, Chris D. Banna, Carolyn M. Radeke, Nikunj Sharma, Mary E. Albertolle, Seng Hui Low, Thomas Weimbs, Carol A. Vandenberg

**Affiliations:** 1 Department of Molecular, Cellular, and Developmental Biology, University of California Santa Barbara, Santa Barbara, California, United States of America; 2 Neuroscience Research Institute, University of California Santa Barbara, Santa Barbara, California, United States of America; National Heart Lung and Blood Institute, UNITED STATES

## Abstract

Cell polarity and precise subcellular protein localization are pivotal to neuronal function. The SNARE machinery underlies intracellular membrane fusion events, but its role in neuronal polarity and selective protein targeting remain unclear. Here we report that syntaxin 3 is involved in orchestrating polarized trafficking in cultured rat hippocampal neurons. We show that syntaxin 3 localizes to the axonal plasma membrane, particularly to axonal tips, whereas syntaxin 4 localizes to the somatodendritic plasma membrane. Disruption of a conserved N-terminal targeting motif, which causes mislocalization of syntaxin 3, results in coincident mistargeting of the axonal cargos neuron-glia cell adhesion molecule (NgCAM) and neurexin, but not transferrin receptor, a somatodendritic cargo. Similarly, RNAi-mediated knockdown of endogenous syntaxin 3 leads to partial mistargeting of NgCAM, demonstrating that syntaxin 3 plays an important role in its targeting. Additionally, overexpression of syntaxin 3 results in increased axonal growth. Our findings suggest an important role for syntaxin 3 in maintaining neuronal polarity and in the critical task of selective trafficking of membrane protein to axons.

## Introduction

Neurons are specialized for directional transfer of information, and require unique sets of membrane proteins in axonal and dendritic regions. Establishment and maintenance of neuronal polarity depends on precise targeting of proteins to functional sites in specialized neuronal subdomains [1–4]. Yet, how neurons achieve a polarized distribution of membrane proteins remains an intriguing and challenging question.

Several mechanisms have been proposed to act at successive stages along the secretory and endocytic pathway to ensure the fidelity of polarized trafficking [1–6]. Early in the biosynthetic pathway, sorting of membrane proteins into distinct cargo vesicles at the Golgi may target them for selective transport to particular regions of the cell, where the vesicles then fuse with the plasma membrane [1, 4, 7]. For some membrane proteins, their polarized distribution depends on their transport in vesicles directly from the Golgi to the correct subcellular site, and involves interactions between transport vesicle, motor proteins and cytoskeleton [8–12]. The axon initial segment and the pre-axonal exclusion zone also contribute to polarized transport, and have been proposed to act as a filter for cargo vesicles, permitting the entry of axonally directed cargoes while restricting the transport of some dendritic cargoes [13–16]. Polarized trafficking of some proteins takes place further downstream through the processes of selective endocytosis or transcytosis, which results in removal of the protein from the site at which it is initially inserted into the membrane, followed by sorting in endosomal compartments, and redirection to its final membrane destination [3, 17, 18]. Scaffolding molecules also may concentrate proteins locally, and membrane barriers in the axonal initial segment may restrict lateral movement, thereby maintaining protein locales [13, 19–21].

An additional mechanism, which we examine here, involves selective recognition between transport vesicles and the target membrane. In this model of protein polarization, cargo vesicles may be transported throughout the cell, but recognize and fuse only with the correct target membrane [8, 17]. This mechanism plays a key role in polarized epithelial cells where distinct SNARE (soluble *N*-ethylmaleimide sensitive factor attachment protein receptor) proteins on apical and basolateral membranes coordinate the selective targeting of transport vesicles to these sites [22–27].

SNARE proteins are the essential components that drive intracellular membrane fusion, which occurs when vesicle SNAREs (v-SNAREs) on the vesicle or donor membrane associate with target SNAREs (t-SNAREs) on the target membrane [28–30]. The fusion complex includes one t-SNARE of the syntaxin (Stx) family, and 2–3 cognate SNAREs [28, 30]. Importantly, different SNARE complexes are localized to different intracellular compartments and are associated with fusion events between different membrane compartments [28]. Among the neuronal plasma membrane syntaxins, Stx1 is the primary form involved in calcium-regulated fusion of synaptic vesicles leading to neurotransmitter release, while Stx2-4 function in additional plasma membrane fusion events [31]. In polarized epithelial cells Stx3 and Stx4 are differentially localized to the apical and basolateral domains, respectively. Stx3 is involved in the selective fusion of post-Golgi carriers with the apical plasma membrane, whereas Stx4 participates in maintenance of basolateral cell polarity [22–27, 32–35]. Whether and how Stx3 and Stx4 may be involved in polarized trafficking in neurons is not clearly understood. Studies in PC12 cells found that Stx3 is involved in neurite outgrowth and suggested a role in axonal trafficking [36]. It has been reported that Stx4 localizes to dendrites and is involved in activity-dependent trafficking of endocytic vesicles to the postsynaptic membrane [37]. Stx4 also was implicated in postsynaptic AMPA receptor trafficking linked with homeostatic plasticity, while Stx3B has been associated with AMPA receptor trafficking in long term potentiation [38, 39]. Despite an established role for SNARE proteins in the mechanics of fusion, their contribution to the specificity of neuronal protein targeting and their role in neuronal polarity are still incompletely understood [28, 29, 40].

Here we investigate the involvement of Stx3 in axonal targeting of membrane proteins. We show that Stx3 and Stx4 are polarized in neurons, with Stx3 trafficking to the axonal and Stx4 to the somatodendritic plasma membrane. We find that the same motif that is required for apical targeting of Stx3 in epithelial cells is also required for axonal targeting in neurons suggesting that a conserved targeting mechanism exists in these cells. Disruption of axonal targeting of Stx3 leads to mislocalization of axonal membrane cargos indicating that Stx3 participates in polarized protein trafficking in neurons. These results provide intriguing insights into the mechanisms of neuronal cell polarity and a link between Stx3 and the targeting of axonal proteins.

## Materials and Methods

### Plasmid and viral constructs

Syntaxin constructs for expression in neurons were based on human Stx3 (homologous to rat Stx3A) or Stx4, which were incorporated into a modified pcDNA4-TO/myc_2_-His (Invitrogen) expression vector containing two carboxyl terminal myc tags followed by a His_6_-tag [23]. Mutant Stx3 construct Stx3Δ38 (lacking the N-terminal 38 amino acids), and triple point mutant Stx3_AAA_ (F31A, D33A, E34A) were subcloned similarly. As previously described, phenotypes for each of the single point mutations F31A, D33A, and E34A resulted in apical mistargeting of Stx3 in MDCK cells [23]. Stx3 and Stx4 constructs in adenovirus with C-terminal myc_2_-His tags were described previously [41], and green fluorescent protein (GFP) adenovirus also was used. Adenoviruses containing mutant Stx3 (Stx3Δ38 and Stx3_AAA_) were constructed in the AdEasy adenoviral system according to manufacturer’s protocols (Stratagene-Agilent, Santa Clara, CA).

Full-length chicken NgCAM-GFP, NgCAM-mCherry and human transferrin receptor (TfR)-GFP constructs contained a carboxyl terminal GFP or mCherry in pJPA7 and pJPA5 [17], and rat neurexin 1α (Nrxn) had an N-terminal GFP [42]. In some experiments, neurons were cotransfected with tdTomato (in pJPA5) or with GFP (pEGFP-C1, Invitrogen).

Small hairpin RNAs targeted to Stx3 (shStx3) or nontarget sequence (shNontarget) were subcloned into pAd shRNA/H1 plasmid that also expresses eGFP in order to identify transfected cells. Three shStx3 plasmids were generated (#14: CGAGGCTCAACATCGACAA; #15: GCTAAGAAACTCTACAGTA; #16: GGCTCGAAAGAAATTGATA), along with a nontarget (control) shRNA: actaccgttgttataggtg that has limited sequence homology to known rat genes. Note that there are two Stx3 splice variants in rat, Stx3A and 3B that differ only in their C-terminus and are identical in other regions including the FMDE motif. For knockdown studies both rat Stx3A and 3B were targeted: two of the shStx3, #14 and #15, encode sequences specific to both Stx3A and Stx3B isoforms, and #16 targets specifically the Stx3A isoform. In most neuronal knockdown experiments, a cocktail of all 3 shStx3 plasmids was used. The efficiency of knockdown for rat Stx3 by the shRNAs was tested by coexpression in Cos-1 cells with plasmids encoding rat Stx3A or rat Stx3B, which were obtained by PCR, sequenced, and subcloned into pcDNA4-TO/myc_2_-His.

### Antibodies

Antibodies were used at the following dilutions and obtained from the following sources: mouse monoclonal anti-Stx1A (1:1,000), rabbit polyclonal anti-Stx3 (1:250), and rabbit polyclonal anti-Stx4 (1:250; Synaptic Systems, Göttingen, Germany), mouse anti-NgCAM 8D9, which recognizes an extracellular NgCAM epitope (1:5 of hybridoma supernatant; Developmental Studies Hybridoma Bank, Iowa City, IA), mouse anti-c-myc 9E10 (1:500; Santa Cruz Biotechnology, Santa Cruz, CA), rat anti-c-myc JAC6 (1:1,000) and rabbit anti-GFP (1:2,000; Abcam, Cambridge, MA), mouse anti-glyceraldehyde-3-phosphate dehydrogenase (GAPDH, 1:500; Chemicon, Billerica, MA), mouse anti-MAP2 (1:500; Sigma-Aldrich, St. Louis, MO), and mouse anti-Tau (Tau-5, 1:1,000; Invitrogen, Grand Island, NY). Alexa Fluor-conjugated secondary antibodies that were highly cross absorbed against multiple species (1:300), and Alexa 488-conjugated rabbit polyclonal anti-GFP (1:1,000) were from Molecular Probes (Invitrogen). Cy3-conjugated secondary antibodies (1:300) were from Jackson ImmunoResearch Laboratories (West Grove, PA). For Western blotting, Alexa 680-conjugated secondary antibodies (1:5,000) were from Molecular Probes and IRDye 800nm-conjugated secondary antibodies (1:5,000) were from Rockland (Gilbertsville, PA).

### Cell culture, transfections and infections

All animal procedures conformed to National Institutes of Health Guidelines for the Care and use of Laboratory Animals and were approved by the Institutional Animal Care and Use Committee of the University of California Santa Barbara. Euthanasia of pregnant rats was performed by CO_2_ inhalation, followed by caesarean section and decapitation of embryos. Primary hippocampal cultures were prepared from embryonic day 19 rat brains with modifications [43]. Briefly, neurons were dissociated by trituration after trypsin and DNase I digestion, and plated on coverslips coated with poly-D-lysine and laminin at a density of 20,000 cells/well in 24-well dishes. Cells were plated in MEM supplemented with 5% FBS, glutamine, B27 (Gibco Invitrogen), penicillin and streptomycin. After 24 h, the medium was replaced by serum-free Neurobasal medium supplemented with B27, GlutaMAX, penicillin and streptomycin. Cultures were treated with 0.5 μM AraC at 5 days in vitro (DIV). Neurons were maintained up to 32 days in culture. For trafficking studies, neurons were transfected with plasmids using the calcium phosphate precipitation method at DIV8 and fixed 10–12 hrs later [44]. For axonal branching and length studies, neurons were transfected at DIV2 and fixed 48 h later. In experiments using more mature neurons at ≥ DIV15, cells were infected with adenovirus constructs by addition of virus to the culture medium, and the cells were fixed 8–24 h later. Use of short expression time after transfection or infection allowed optimal detection of polarized distribution of the expressed proteins. Cos-1 cells and NRK (normal rat kidney) cells (ATCC, Manassas, VA) were cultured as described previously [45], and Cos-1 cells were transfected using FuGENE 6 (Roche, Indianapolis, IN).

### RT-PCR and immunoblotting

For RT-PCR reactions, total RNA was isolated from DIV15 cultured hippocampal neurons and NRK cells, and reverse transcribed and amplified using Stx-specific primers as described previously [34]. For immunoblotting, total membrane fractions were harvested from DIV15 cultured hippocampal neurons and confluent NRK cells by scraping the cells into HEES buffer (20 mM Hepes, 5 mM EGTA, 5 mM EDTA, 320 mM sucrose, pH 7.4) containing Complete protease inhibitors (Roche) and were then homogenized on ice by repeated passage through a 25-gauge needle. After a brief centrifugation at 4°C (100 *x g*, 10 min) to remove intact cells and nuclei, the supernatant was saved on ice. The pellet was rehomogenized and centrifuged, as before, and supernatants from the centrifugations were pooled. Membranes were recovered by centrifugation at 100,000 x g, for 30 min at 4°C and resuspended in HEES buffer. Bradford assays were performed to measure protein concentration. Hippocampal lysates from adult rats were prepared as described previously [45]. Proteins were separated by 12% SDS-polyacrylamide gel electrophoresis, transferred to nitrocellulose and incubated in Odyssey blocking buffer (Li-Cor, Lincoln, NE). Affinity-purified Stx3 and 4 antibodies (Synaptic Systems) showed cross-reactivity with related syntaxin species (Stx 1–4). To eliminate this cross-reactivity, Stx3 antibodies were preabsorbed with GST-Stx 1, 2, and 4 by incubating the antibodies with a mixture of bacterial lysates that expressed these various GST-Stx antigens as described previously [46]. Similarly, Stx4 antibodies were preabsorbed against GST-Stx 1, 2, and 3. Afterwards, the preabsorbed anti-Stx3 or anti-Stx4 antibodies were used to probe Western blots containing the various cell membrane lysates followed by detection using secondary antibodies conjugated to either Alexa Fluor 680 or IRDye 800. Protein bands were visualized and quantified with an Odyssey infrared imager (Li-Cor).

### Trafficking studies and immunofluorescence labeling

Trafficking and localization studies of Stx3, Stx3 mutants and Stx4 in cultured rat hippocampal neurons were performed following expression of Stx constructs with adenovirus or transfection. For surface labeling, cells were rapidly fixed without permeabilization by incubation for 4 min at room temperature with 4% paraformaldehyde, 4% sucrose in phosphate buffered saline (PBS) that was prewarmed to 37°C. Cells were then washed with PBS, and blocked in PBS, 3% BSA, 1% donkey serum, 1% goat serum for 30 min. Primary antibodies diluted in blocking solution were added for 35 min, followed by incubation with fluorescently-conjugated secondary antibodies. For internal labeling, cells were subsequently permeabilized in blocking solution containing 0.2% Triton X-100 for 1 h. A second round of primary antibodies was applied for 45 min. Primary antibodies were detected by incubation with the appropriate fluorescent-conjugated secondary antibodies for 45 min. Coverslips were mounted in Prolong (Invitrogen). Images shown are representative of several hundred neurons for each experimental condition. Surface and internal syntaxins were labeled using rat and mouse anti-myc antibodies before and after membrane permeabilization.

For trafficking studies with NgCAM, TfR or Nrxn protein cargos, neurons were cotransfected with the cargo protein tagged with GFP, together with Stx3, Stx4 or mutant Stx3_AAA_ or Stx3Δ38. After overnight expression, surface NgCAM was detected with anti-NgCAM antibody, 8D9, followed by permeabilization and labeling of total Stx with anti-myc antibody and total NgCAM with anti-GFP antibody. A similar approach was used to measure trafficking of TfR or Nrxn, using an unconjugated rabbit anti-GFP antibody to detect surface TfR or Nrxn, followed by permeabilization and labeling of total Stx with anti-myc and internal TfR or Nrxn with anti-GFP antibody conjugated to Alexa 488.

For live cell labeling, live neuronal cultures were incubated with primary antibodies in culture medium for 20–30 min at 37°C, quickly but gently washed in 37°C culture medium, and fixed in 4% paraformaldehyde, 4% sucrose in PBS prewarmed to 37°C. Further labeling procedures were carried out as described above for surface labeling.

To investigate polarized distribution of endogenous cytoskeletal proteins Tau and Microtubule-associated protein 2 (MAP2), young neurons were cotransfected with Stx constructs and GFP, and 2 days later (DIV 4–5) were fixed, permeabilized, and labeled for Tau, MAP2, myc-Stx constructs and GFP.

### Image quantification

Images of transfected neurons were captured on a Zeiss Axiovert 200M microscope with either Plan Neofluor 40x/1.3, Plan Apochrome 20x/0.8 or Fluor 10x/0.5 objectives. Only neurons expressing low to moderate amounts of Stx3 or Stx4 were chosen for analysis to minimize overexpression artifacts. Image analysis was performed using routines written for Axiovision 4.5 software (Zeiss, Thornwood, NY). Images were displayed using Photoshop, with gamma correction only of soluble GFP images where needed to visualize cell morphology.

#### Axonal length and branching

Axonal length and branching were measured for DIV4 neurons that were plated at sufficiently low density that axonal processes did not overlap significantly with neighboring neurons. Young neurons were chosen for this study because their axons could be clearly identified and measured. For consistency, all measurements were made using the image of the GFP-filled neuron, and syntaxin expression was confirmed in each neuron by anti-myc labeling. The length of the longest axon branch was measured from a line traced along the longest axon branch from the cell body to axon tip for each neuron. Total axonal length was similarly measured by tracing lines throughout the entire extent of axonal processes for each neuron. Axonal branching was scored and categorized into either long or short lengths by counting the number of branch points that were followed by a long (≥ 40 μm) or short (< 40 μm) segment of axon.

#### Axonal/dendritic polarity index ratio

Surface NgCAM, TfR, Nrxn and Stx labeling was quantified by measurement of average axonal to dendritic polarity ratios [17, 47]. Briefly, one-pixel wide lines were drawn along the dendrites and axon from images acquired with a Plan Apochrome 20x/0.8 objective. Mean fluorescent pixel intensities of axonal and dendritic lines were measured and corrected for background. An average axon:dendrite polarity index (PI) ratio was calculated for each neuron by dividing the mean axonal fluorescent intensity by the mean dendritic intensity. Theoretically, an axon:dendrite ratio of 1 indicates a uniform protein distribution. In practice, we considered PI values ranging from 0.7 to 1.5 as indicative of a uniform labeling pattern. Mean PIs of >1.5 were designated axonal and ratios of <0.7 were designated somatodendritic. Data are presented as mean values ± SEM. Statistical analysis was conducted by one-way ANOVA, and differences between conditions were determined with a prior contrast test (SPSS software). For experiments comparing only two conditions, statistical differences were determined by Student’s t-test.

## Results

### Stx3 is targeted to axons, and Stx4 to dendrites in hippocampal neurons

**Fig 1:**
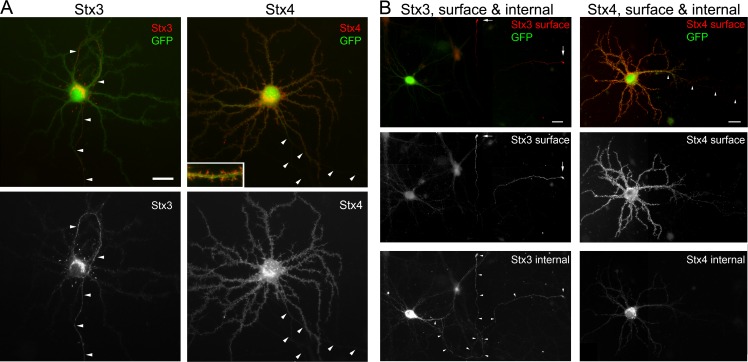
Stx3 is polarized to axons and axon tips, and Stx4 to dendrites in hippocampal neurons. (A) Localization of Stx3 and Stx4 expressed in mature neurons. Neurons were coinfected with adenoviruses expressing either myc-Stx3 (left) or myc-Stx4 (right), and GFP. Total Stx expression was determined by immunolabeling after membrane permeabilization using anti-myc antibody, and cell morphology was highlighted with anti-GFP antibody. Neurons are representative of several hundred cells. Bar, 20 μm. Inset shows magnified view of a region of dendrite (same bar for inset, 8 μm). (B) Surface and internal labeling of Stx3 and Stx4 was determined with rat anti-myc antibody, followed by membrane permeabilization, and labeling for internal Stx3 (mouse anti-myc) and GFP. Surface Stx3 was prominent in axon tips, whereas Stx4 was localized to dendritic spines, shaft and cell soma. Images are montages of 2–4 images taken at identical exposure settings. Arrowheads mark the axon, which was identified in the GFP image; arrows denote axon tips. Bars, 20 μm.

To determine whether plasma membrane SNAREs could contribute to the polarized trafficking of membrane proteins in neurons, we investigated the distribution pattern of the plasma membrane syntaxins 3 and 4 in mature hippocampal neurons by examining the distribution of myc-tagged Stx3 and Stx4 and GFP using transient adenovirus-mediated expression (Fig 1A). We found that Stx3 was polarized to the axon (Fig 1A and S1 Fig, arrowheads), which was identified morphologically, and by the presence of the axonal marker Tau and absence of the dendritic marker MAP2 (S1 Fig). Stx3 was abundant in axon tips (see below), and was nearly absent in the dendrites. Labeling also was sometimes observed at the tips of neurites surrounding the cell body, which appeared to be sprouting tips of immature neurites. In sharp contrast to the axonal localization of Stx3, Stx4 localized to the cell body and dendrites, with labeling extending to the distal portions of the dendritic branches (Fig 1A). Dendritic spines were clearly labeled (Fig 1A inset), in agreement with a proposed role in trafficking in postsynaptic membranes [37, 39]. Polarized expression of Stx3 and Stx4 was consistently observed at all ages of neurons tested, from early (DIV3) through mature (DIV32).

The surface expression of Stx3 and Stx4 was next investigated using a surface and internal labeling protocol. Because wild-type Stx3 and Stx4 lack an extracellular domain, we used double myc epitope tags inserted downstream of the C-terminal transmembrane domain to enable surface detection. Previous studies have demonstrated that polarized targeting in epithelial cells is unaffected by the myc tag [23, 26, 27]. By labeling cells at early times (< 16 hrs) after adenovirus infection, we found that plasma membrane Stx3 was first detected at axon tips, and not at the proximal axonal region (Fig 1B and S2 Fig), suggesting that it is preferentially inserted at or near the growing tip. Internal labeling revealed that intracellular Stx3 was present in the soma and throughout the axon, and was abundant at the axon tips (Fig 1B). Comparison of surface Stx3 in axons vs. dendrites is depicted for representative mature neurons with well-developed dendritic spines in S2 Fig, and showed that Stx3 was significantly more abundant on the axonal surface than on dendrites. Surface Stx4, on the other hand, was detected prominently on dendritic spines and shafts and the cell soma (Fig 1B), with intracellular Stx4 residing primarily in the soma. This polarized pattern of cell surface Stx3 at the tips of axons suggests that this SNARE protein could potentially participate in protein targeting to axonal membrane domains.

### Stx3 and Stx4 are expressed in hippocampus

To examine the expression of endogenous Stx3 and 4 in hippocampal neurons, we employed RT-PCR and immunoblotting, and demonstrated that endogenous Stx3 and 4 are present in hippocampal cultures and in adult rat hippocampus (S3 Fig). Cultured neurons expressed Stx3A, Stx3B and Stx4 transcripts (S3A Fig), consistent with previous findings [38, 48, 49]. Immunoblotting demonstrated that Stx3 and Stx4 protein are present in hippocampal cultures and adult rat hippocampal tissue (S3B Fig). Although we tested a variety of Stx antibodies, we did not find Stx3 or Stx4 antibodies that were sufficiently sensitive for reliable immunocytochemistry so an alternative approach utilizing siRNAs to knock down Stx3 was chosen to investigate the role of endogenous syntaxins in hippocampal neurons (below).

### Stx3 promotes axonal outgrowth

**Fig 2:**
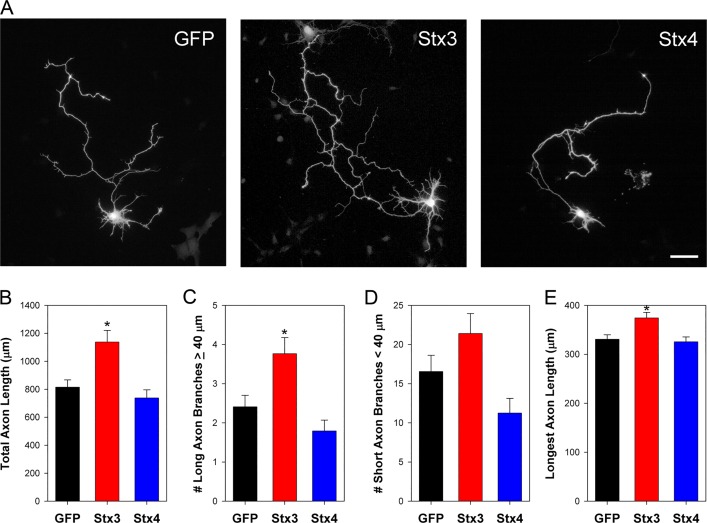
Stx3 overexpression results in increased axonal length and branching. (A) DIV4 neurons coexpressing GFP in the absence (left) or presence of Stx3 (middle) or Stx4 (right). Stx3-expressing neurons had longer axons, with no difference in dendrites or in axonal width observed between conditions. To highlight morphology, images show GFP labeling of representative neurons. Scale bar, 50 μm. (B-D) Quantification of neuronal morphology showed that Stx3-expressing cells had increased total axonal length, increased number of long axonal branches, and increased length of the longest primary axon compared to control. Error bars, SEM; N = 33–35 in B, C and D; N = 183–315 in E; * P≤ 0.01. This experiment was repeated independently three times.

Given that SNAREs mediate vesicle fusion, it seemed possible that an increase in expression of Stx3 could stimulate expansion of the axonal membrane and axonal growth. Neurons (DIV2) were cotransfected with GFP and either Stx3 or Stx4, and axonal morphology was measured two days later. When Stx3 was expressed, neurons exhibited an increase in axonal length and arborization compared to the GFP-alone control (Fig 2A). Conversely, axon morphology was maintained in neurons transfected with Stx4. Overexpression of Stx3 resulted in ~40% increase in overall axon length with a significant increase (~50%) in the number of long axonal branches and a modest but significant increase (10–15%) in the length of the longest axonal process (Fig 2B–2E). No statistically significant difference in axonal growth or extent of axonal branching was observed with expression of Stx4. These results are consistent with previous studies showing that siRNA-mediated knockdown of Stx3 caused inhibition of neurite outgrowth, and the proposal that Stx3 may be involved in vesicle fusion in the axon, resulting in expansion of the axonal membrane and growth [36].

### N-terminal targeting motif of Stx3 is required for axonal trafficking

**Fig 3:**
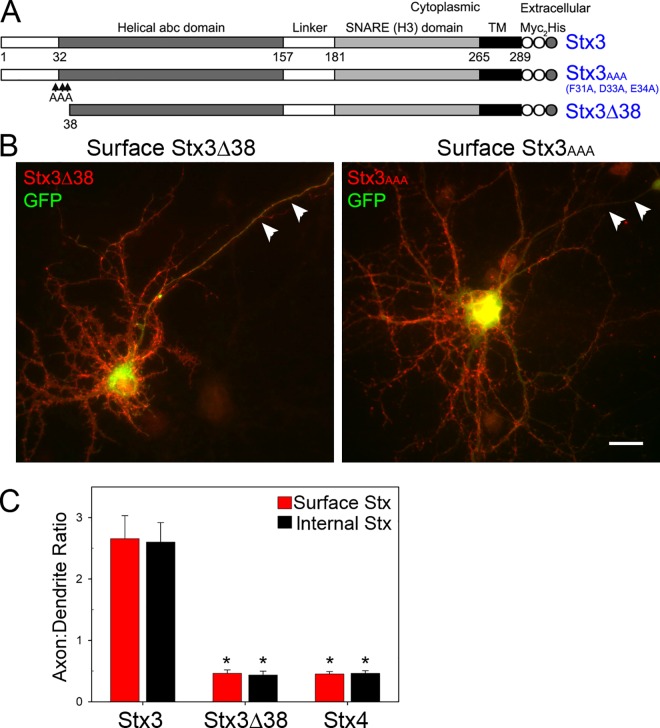
The N-terminal FMDE motif of Stx3 is required for axonal targeting. (A) Stx3 wild-type and mutant constructs are schematically depicted. Tandem myc epitope tags are present at the C-terminus (extracellular domain) to facilitate surface labeling [23]. Stx3Δ38 contains a 38 amino acid N-terminal truncation. In Stx3_AAA_, three residues of the FMDE motif of Stx3 were changed to alanine as indicated by ‘AAA’. (B) Mutant Stx3 constructs lacking the FMDE motif are mistargeted to the somatodendritic, as well as axonal plasma membrane. Neurons were coinfected with adenoviruses expressing Stx3Δ38 or Stx3_AAA_ and GFP, and were live surface labeled with anti-myc antibody (red). Arrowheads denote axon. Bar, 20 μm. (C) Quantification of axon:dendrite polarity index for surface (red bars) and internal (black bars) Stx3, Stx3Δ38 and Stx4 in mature hippocampal neurons. PI ratios for Stx3Δ38 and Stx4 were significantly reduced compared to Stx3. Error bars, SEM; N = 14–18; * P < 0.0001.

The repertoire of axonal targeting motifs identified thus far is diverse [2, 3, 19, 50, 51]. Studies have uncovered an N-terminal motif (FMDE) required for the apical localization of Stx3 in epithelial cells [23, 24]. To determine whether this motif influences Stx3 polarity in neurons, we used two mutant constructs that disrupt epithelial apical targeting: a triple alanine point mutation (Stx3_AAA_) within the FMDE motif, and a truncation of the N-terminal 38 amino acids (Stx3Δ38) (Fig 3A). Both Stx3Δ38 and Stx3_AAA_ displayed striking mislocalization in neurons, with labeling in both somatodendritic and axonal regions (Fig 3B). Quantification of the axon:dendrite ratio, the polarity index, showed that surface and internal Stx3 is polarized to the axon (Fig 3C and S2 Fig), whereas mutant Stx3Δ38 is mislocalized, and Stx4 is localized to dendrites (Fig 3C). Note that quantification of the polarity index measures average axon:dendrite surface labeling over the entire length of axon and dendrite. Since Stx3 tends to be preferentially localized near the axon tips, this underestimates the polarized distribution of Stx3 at the axon tips. These results indicate that a conserved N-terminal targeting motif is required for Stx3 axonal targeting, and suggests that a similar mechanism of polarized targeting is employed by epithelial cells and neurons.

### Mistargeting of Stx3 disrupts axonal localization of NgCAM, an axonal cargo protein, but not somatodendritic localization of TfR

According to the SNARE model of membrane fusion, v-SNAREs in transport vesicles recognize and bind to cognate t-SNAREs on the target plasma membrane, thereby initiating membrane fusion and delivery of cargo membrane proteins to the plasma membrane. If the SNARE fusion machinery also contributes to the *specificity* of vesicle targeting, then trafficking of cargo proteins should be defined by the location of the plasma membrane t-SNAREs. To test this hypothesis directly, we investigated whether the localization of Stx3 would influence the trafficking of axonal cargo proteins. We examined a well-known axonal cargo protein, NgCAM, which has been previously demonstrated to be intracellular in both the axonal and dendritic compartments but polarized to only the axonal surface [8, 17, 47], and asked whether mistargeting of Stx3 could result in misdirected localization of NgCAM.

**Fig 4:**
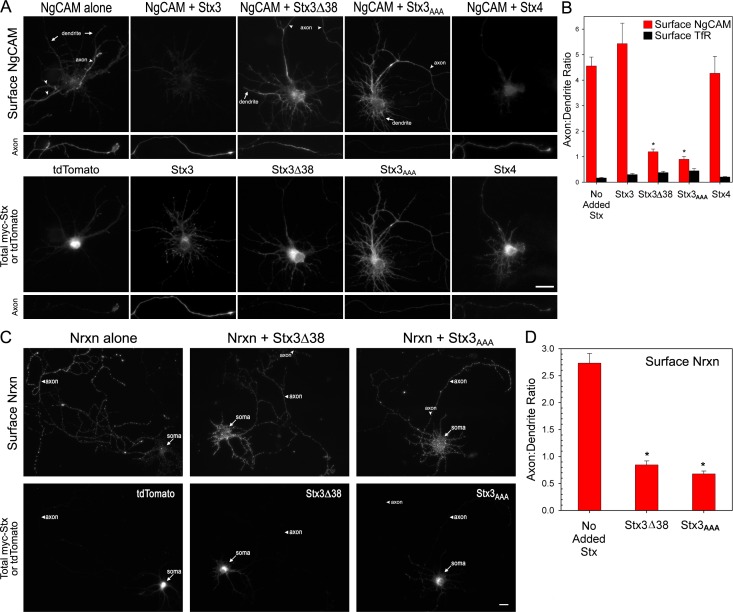
Mistargeting of Stx3 disrupts axonal polarization of NgCAM and neurexin 1 but not dendritic polarization of transferrin receptor. (A) NgCAM is targeted to the axon surface when expressed alone (with tdTomato) or in the presence of wild type Stx3, but when cotransfected with Stx3Δ38 or Stx3_AAA_, NgCAM is mislocalized to the somatodendritic region together with mutant syntaxin. Neurons were cotransfected with NgCAM-GFP in the absence or presence of Stx3, Stx3Δ38, Stx3_AAA_, or Stx4. After overnight expression, the cells were surface labeled for plasma membrane NgCAM (8D9 antibody), and then were permeabilized and internally labeled with anti-myc to detect total syntaxin, and anti-GFP to label total NgCAM. Arrows indicate dendrite, arrowheads denote axon; axon segments are at or near the axon tip; bar, 20 μm. (B) Quantification of axon:dendrite polarity index for surface NgCAM or TfR coexpressed with Stx constructs. NgCAM polarity index was decreased in neurons coexpressing Stx3Δ38 or Stx3_AAA_, whereas TfR, a somatodendritic cargo, was somatodendritic under all conditions tested (see images S4 Fig). Error bars, SEM; N = 20–21 for NgCAM, 13–14 for TfR; * P ≤ 0.0001. (C) Nrxn is targeted to the axon when expressed alone (with tdTomato), but is mislocalized to the somatodendritic region when coexpressed with Stx3 mutants. Bar, 20 μm. (D) Axon:dendrite polarity index for surface Nrxn was reduced when Nrxn was coexpressed with Stx3Δ38 or Stx3_AAA_ compared to control. Error bars, SEM; N = 26–30; * P<0.0001.

Indeed, we found that surface NgCAM was properly localized to the axon in the presence of wild type Stx3, but when cotransfected with Stx3 mutants, both NgCAM and the Stx3 mutants were markedly mislocalized to the somatodendritic region. When NgCAM was expressed alone, surface NgCAM was strongly polarized to the axon, concentrated at the axon tips (Fig 4A). Upon coexpression with wild type Stx3, NgCAM axonal polarity was maintained, and substantial colocalization between surface NgCAM and total Stx3 was observed (Fig 4A). However coexpression with Stx3Δ38 or Stx3_AAA_ mutants led to coincident mislocalization of both surface NgCAM and mutant Stx3 to the somatodendritic region and a reduction of surface NgCAM in the axon (Fig 4A and 4B). In contrast, Stx4 showed minimal overlap with surface NgCAM, and no effect on surface NgCAM polarity, suggesting that Stx4 does not influence the targeting of this axonal cargo.

To quantify these data, we measured the degree of polarization for surface NgCAM by determining the average axon:dendrite ratio. For control cells expressing NgCAM alone, the ratio was 4.6 ± 0.3, similar to that observed with Stx3 coexpression (5.4 ± 0.8), and indicative of strong axonal polarization (Fig 4B, red bars). However, in the presence of syntaxin mutants Stx3Δ38 or Stx3_AAA_, the axon:dendrite ratio for surface NgCAM decreased substantially to 1.2 ± 0.1 and 0.9 ± 0.1, respectively (Fig 4B, red bars), which reflects values observed for uniformly distributed proteins.

Parallel studies with transferrin receptor (TfR), a somatodendritic cargo, showed that mistargeting of Stx3 did not affect localization of TfR. Under all conditions, surface TfR was highly restricted to the somatodendritic surface regardless of coexpression with various Stx constructs (Fig 4B, black bars, and S4 Fig). These results suggest that the polarized trafficking of vesicles carrying the axon-bound NgCAM cargo, but not the dendrite-bound TfR cargo, depends on axonal polarization of Stx3.

### Mistargeting of Stx3 disrupts axonal polarization of neurexin

To investigate whether polarized targeting of another axonal protein depends on Stx3, we investigated the presynaptic adhesion molecule neurexin 1α (Nrxn). In agreement with previous results [42], we found that Nrxn is well polarized to the axon (Fig 4C and 4D). Cotransfection of Nrxn with either Stx3Δ38 or Stx3_AAA_ resulted in mistargeting of surface Nrxn to the somatodendritic region together with the mutant Stx3Δ38 or Stx3_AAA_, and caused a significant decrease in the surface Nrxn axon:dendrite polarity ratio (Fig 4C and 4D).

### Mistargeting of Stx3 does not affect polarization of cytoskeletal proteins Tau or MAP2

**Fig 5:**
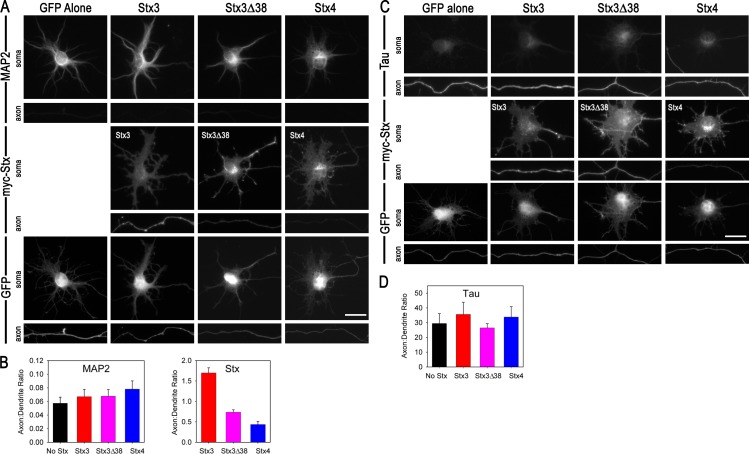
Mistargeting of Stx3 does not alter polarized localization of cytoskeletal proteins MAP2 (somatodendritic) or Tau (axonal). (A) Endogenous MAP2 was labeled in neurons (DIV 4) 2 days after cotransfection with Stx3, Stx3Δ38 or Stx4 together with GFP. (B) Quantification of the axon:dendrite polarity index for endogenous MAP2 and Stx constructs. MAP2 was polarized to the somatodendritic region, and was not significantly different between groups, whereas Stx3 was primarily axonal, Stx3Δ38 was throughout the neurons, and Stx4 was primarily somatodendritic. (C) Endogenous Tau was labeled in neurons (DIV 5) 2 days after cotransfection with Stx3, Stx3Δ38 or Stx4 together with GFP. (D) Quantification of the axon:dendrite polarity index for endogenous Tau. Tau was strongly polarized to the axon, and was not significantly different between groups. Bar, 20 μm.

Since SNARE proteins are involved in cargo delivery via membrane fusion, we predicted that the polarized localization of non-membrane proteins would not be affected by Stx3. To test this, distributions of the cytoskeletal proteins Tau and MAP2 were examined in early neurons where they are characteristically highly polarized to axons and dendrites, respectively. As expected, overexpression of Stx3, Stx3Δ38 or Stx4 did not significantly alter the axonal polarization of Tau (Fig 5C and 5D) or the somatodendritic polarization of MAP2 (Fig 5A and 5B), suggesting that these SNAREs specifically affect the delivery of membrane proteins.

### Expression of shRNA to Stx3 alters axonal polarity of NgCAM

**Fig 6:**
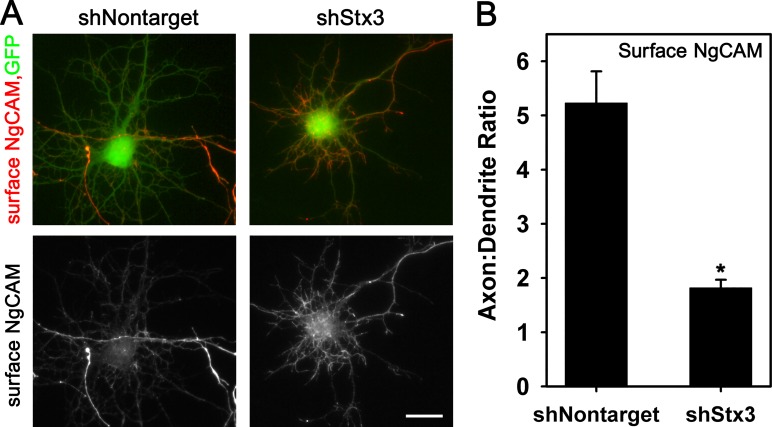
Expression of shRNAs to Stx3 leads to mistargeting of surface NgCAM to the somatodendritic region. (A) Neurons were transiently cotransfected with NgCAM-mCherry together with a pool of shStx3 or with shNontarget as a negative control. Neurons transfected with shRNAs were identified by GFP expression and imaged by immunofluorescence. Bar, 20 μm. (B) The axon:dendrite polarity ratio for surface NgCAM was significantly reduced for cells cotransfected with shStx3 compared to shNontarget. Error bars, SEM; N = 37 for shNontarget, N = 47 for shStx3; * P < 0.0001.

Since our previous data indicated that Stx3 can influence axonal targeting, we next asked if endogenous Stx3 is required for polarized targeting of axonal membrane protein. Using NgCAM as the axonal cargo we investigated the effect of shRNAs that knockdown Stx3. We generated shRNAs for rat Stx3 in plasmids that also express GFP in order to identify transfected cells, and subsequently validated their knockdown efficiency by Western blot in Cos-1 cells (S5 Fig). Pools of the shStx3 plasmids (or a control nontarget shRNA) were expressed with mCherry-NgCAM in hippocampal neurons, and the distribution of surface NgCAM was assessed 22–24 hrs later when NgCAM levels were low and its axonal polarization was optimal in control neurons, although knockdown of Stx3 was still incomplete. Neurons transfected with control nontarget shRNA and NgCAM displayed normal surface NgCAM polarity, with high levels of NgCAM on the surface of the axon and axonal tips, and little NgCAM on somatodendritic surface membranes. (Fig 6A). In contrast, neurons transfected with shStx3 exhibited significant mislocalization of NgCAM to the somatodendritic surface, with more abundant labeling on the surface of the soma and the proximal regions of the dendrites than control neurons (Fig 6A). Often, however, some NgCAM was present on the axonal surface, which may reflect the presence of residual Stx3. The axon:dendrite ratio of surface NgCAM was significantly reduced in cells expressing Stx3 shRNAs (1.8 ± 0.2), compared to the ratio for control neurons expressing nontarget shRNA (5.2 ± 0.6) (Fig 6B). Although Stx3 levels were not measured directly in these individual neurons, the loss of NgCAM polarity upon expression of shRNAs that knockdown Stx3 strongly suggests that endogenous SNARE-mediated cargo delivery plays a key role in determining the specificity of NgCAM axonal trafficking.

## Discussion

Central to the functional specialization of neurons is the proper localization of membrane proteins. By investigating the targeting of axonal and dendritic proteins in hippocampal neurons, our studies indicate that Stx3, a plasma membrane t-SNARE, participates in the targeted delivery of axonal cargos. It has been previously proposed that SNAREs, which control membrane fusion, may play a role in polarized protein targeting to the axon [1], but to our knowledge experimental studies to test the hypothesis have not been previously reported. Current understanding of the contribution of SNAREs to the specificity of protein targeting has been based largely on studies carried out in liposome fusion assays *in vitro*, in epithelial cells, or in neuronal dendrites [22–24, 26, 27, 35, 37–39, 52–55]. Here we show that Stx3 is polarized in neurons. The same protein motif that is required for Stx3 axonal targeting in neurons is also required for its apical targeting in epithelial cells. Disruption of axonal targeting of Stx3 leads to mislocalization of axonal membrane protein cargos NgCAM and neurexin, indicating that Stx3 is involved in polarized targeting of these membrane proteins. Neither a dendritic membrane protein, nor polarized cytoskeletal proteins were affected by manipulation of Stx3, suggesting that Stx3 confers specificity for delivery of axonal membrane cargos. In addition, Stx3 overexpression leads to axonal outgrowth and increased axonal arborization. These results provide evidence that SNARE proteins play a role in the mechanisms involved in axonal targeting of membrane protein and maintenance of neuronal polarity.

Monitoring the appearance of newly synthesized Stx3 on the plasma membrane revealed that Stx3 is selectively inserted at or near the tips of axons. These results are consistent with the report that Stx3 is present in neurites of PC12 cells and growth cones of hippocampal neurons, although its polarized distribution in cells was not evaluated [36]. In the retina, where Stx3 is abundant, studies have shown that it is localized to synaptic terminals and inner segments of photoreceptors, and axonal terminals of bipolar cells [49, 56–58]. Perhaps due to lower abundance in brain, reports have not detected significant differences in pre- and postsynaptic distribution of endogenous Stx3 by immuno-EM [38], and a recent report was unable to find an antibody suitable for labeling brain after testing ten Stx3 antibodies [59]. Consistent with the dendritic localization of Stx4 reported here, Stx4 was found to be enriched on the lateral domain of dendritic spines and required for the fusion of recycling endosomes during elevated synaptic activity [37]. Stx4 also has been implicated in dendrites in AMPA receptor trafficking during homeostatic plasticity, whereas a requirement for Stx3B was reported for AMPA receptor exocytosis to the postsynaptic membrane in long term potentiation [38, 39]. While our studies indicate that Stx3 (homologous to rodent Stx3A) is preferentially trafficked to the axon, we cannot exclude the possibility that low levels of Stx3 (or Stx3B) may be involved in exocytic events in dendrites. Overall, our findings suggest that Stx3 is targeted to axon tips where it is well positioned to mediate fusion of cargo vesicles to deliver axonal membrane proteins.

Proof that any protein confers targeting specificity requires the demonstration that when that protein is moved to a different location, then targeting specificity is reprogrammed to the new site. In order to test whether mislocalized Stx3 could relocate the site of cargo vesicle delivery, we first sought to identify the axonal targeting motif of Stx3. Axonal targeting of membrane proteins is achieved by a diverse array of targeting motifs rather than a more limited number of consensus sequences found for dendritic targeting [2, 3, 19, 50, 51, 60]. Our studies showed that an N-terminal domain centered around a conserved FMDE motif is involved in polarized targeting of Stx3 in neurons, similar to its role in targeting Stx3 to the apical domain of MDCK epithelial cells [23, 24, 32]. Stx1, which is involved in neurotransmitter vesicle release at synaptic terminals, also contains a conserved FMDE motif [23], and we speculate that the same motif may be involved in axonal targeting of Stx1. While the molecular mechanisms underlying how cells sort and localize Stx3 remain to be determined, these findings indicate that similar protein sorting pathways are shared by neurons and epithelial cells.

It has been well established that assembly of matching v- and t-SNAREs drives membrane fusion, but the degree to which SNAREs contribute to specificity in vesicle targeting is controversial. In vitro binding studies have indicated that SNAREs assemble into core complexes promiscuously in solution [61, 62]. On the other hand, membrane-based fusion assays showed that SNARE pairing can be highly specific, suggesting that fusion specificity is achieved when SNAREs are incorporated into membranes [53–55, 63].

By investigating the trafficking of axonal cargo proteins NgCAM and neurexin, our studies support a model in which SNAREs play a role in defining the specificity of vesicle targeting in neurons. NgCAM, the chicken homologue of L1, is a neural cell adhesion molecule that accumulates preferentially on the axonal plasma membrane, and thus is well suited for analysis of polarized protein targeting in neurons [17, 47]. Neurexins are axonally localized proteins that are targeted to the presynaptic terminal and mediate signaling and adhesion with their post-synaptic partner neuroligin [42]. Mechanisms underlying transport of nascent NgCAM-laden vesicles from the Golgi to the axon have become increasingly well defined. Vesicles carrying NgCAM traffic in intracellular compartments in both axons and dendrites [8], and studies indicate that NgCAM reaches the axonal membrane via selective fusion [17]. A transcytosis route has been proposed for NgCAM trafficking, wherein NgCAM is transported first to the somatodendritic membrane, then is endocytosed and finally NgCAM-containing vesicles fuse with the axonal membrane [47, 64]. Our data suggest that Stx3 influences the final critical targeting decision of these axonal proteins. Mislocalization of Stx3 mutants to the somatodendritic region causes enrichment of NgCAM and Nrxn on the somatodendritic membrane, possibly by increasing the ability of their transport vesicles to fuse with the somatodendritic membrane in the presence of mislocalized Stx3. We note that when Stx3 was mislocalized, NgCAM and neurexin became similarly mislocalized, although they appeared to be somewhat less dendritically localized than the Stx3 mutant, perhaps due to the presence of endogenous wildtype Stx3 in the neurons. Partial knockdown of Stx3 also decreased the axonal polarization of NgCAM, consistent with the hypothesis that fusion of NgCAM transport vesicles to the axonal plasma membrane was reduced. Under these conditions, NgCAM transport vesicles may be diverted to other non-specific trafficking pathways. Additionally, background levels of somatodendritic NgCAM prior to transcytosis may contribute to the observed decrease in the polarity index of NgCAM with Stx3 knockdown.

Axonal outgrowth, a process involving an increase in the surface area of the plasma membrane, also has been suggested to involve SNARE-mediated fusion of vesicles with the plasma membrane [65]. Consistent with this idea, we found that overexpression of Stx3, localized in the axon, resulted in a substantial increase in the overall axonal membrane length and arborization. These findings are consistent with studies showing that fatty acids and growth factors that stimulate neurite outgrowth also promote interaction of Stx3 and the t-SNARE protein SNAP-25 [36]. Importantly, RNAi-mediated Stx3 knockdown inhibited neurite outgrowth and abrogated neurite branching, suggesting that Stx3 plays a key role in these functions [36]. Complementary work showed that neurite outgrowth was inhibited when dorsal root ganglion neurons were treated with botulinum neurotoxin C, which cleaves and inactivates components of the SNARE complex including Stx3 [66, 67]. Similarly, in photoreceptors Stx3 and SNAP-25 have been implicated in membrane expansion in disc biogenesis [56, 58]. In contrast, it was reported that Stx1A overexpression had either no effect or inhibited neurite extension [68, 69], suggesting specific roles for Stx3 in protein and membrane trafficking and for Stx1 in neurotransmitter release. Together these data suggest that SNARE-mediated membrane events contributes to the polarized delivery of lipids and membrane proteins to axons, thereby promoting both axonal growth and polarized protein targeting.

The binding partners of Stx3 in neurons have not yet been identified. However, it has been demonstrated that NgCAM is transported in neurons in vesicles containing the v-SNARE protein tetanus neurotoxin-insensitive VAMP (TI-VAMP/VAMP7) [70], and previous reports showed that TI-VAMP is enriched at the leading edge of growth cones [71]. In addition, disrupting the interactions between TI -VAMP and SNAP-25 inhibited neurite outgrowth, while overexpression of an N-terminal deletion mutant of TI-VAMP stimulated neurite outgrowth [72]. In epithelial cells, TI-VAMP forms a complex with Stx3 and colocalizes with it on the apical plasma membrane [73]. Additionally, in epithelial cells Stx3 functions in conjunction with Rab GTPases and synaptotagmin-like proteins to control vesicle tethering and fusion to the apical surface [74]. In neurons the SNARE proteins also may interact with regulatory proteins that are involved in vesicle tethering and membrane identity to provide multiple layers of targeting specificity [74–77]. It will be informative in future studies to determine whether these or other proteins act together with Stx3 in the biogenesis of the axonal membrane during development and in the maintenance of axonal polarity. In addition, it will be of interest to determine whether SNAREs are generally involved in targeting of axonal membrane proteins, or whether they primarily influence a specific subset of proteins.

Overall our results suggest that Stx3 participates in polarized axonal targeting of NgCAM and Nrxn, possibly by conferring specificity for selective fusion of cargo vesicles to the axonal plasma membrane. Fidelity in sorting and transport of membrane proteins occurs at multiple levels. We suggest that SNARE-mediated specificity operates in concert with upstream mechanisms for protein sorting and vesicle tethering including selective cytoskeletal transport, and selective endocytosis [11, 18, 75] to orchestrate neuronal polarity, and is one of several mechanisms utilized by cells to ensure targeting to specific subcellular domains.

## Supporting Information

S1 Fig Stx3 is trafficked to axons. (A) Total Stx3 in neuron infected with adenoviruses encoding Stx3 and GFP (labeled in permeabilized neuron with anti-myc and anti-GFP, respectively) is shown for neuron co-labeled with anti-Tau to identify axons. (B) Total Stx3 expression is similarly shown for a neuron co-labeled with anti-MAP2 to identify dendrites. Arrowheads indicate axon. Bar, 25 μm.
(TIF)

S2 Fig Axonal surface expression of Stx3: comparison in axon and dendrites of mature neurons. (A-D) Stx3 surface expression is shown for representative neurons (DIV 32) 1 day after infection with adenoviruses encoding Stx3 (surface labeled with anti-myc, then Alexa 647 secondary antibody; magenta) and GFP to highlight cell morphology (labeled with anti-GFP-Alexa 488, green). Boxed regions are enlarged at right to compare intensity and distribution of surface Stx3 and soluble GFP in axons and dendrites. Stx3 surface labeling was bright in axons, particularly at axon tips, and was much lower in dendrites and dendritic spines. Neurons A and C are single images, neuron B is a montage of 2 images with identical exposure times, and neuron D shows proximal and distal regions with identical exposure times. Arrow indicates axon hillock. Bar, 10 μm (images left), 5 μm (insets at right).
(TIF)

S3 Fig Stx3 and Stx4 are endogenously expressed in rat hippocampus and neuronal cultures. (A) mRNA was amplified by RT-PCR using primer pairs that distinguish Stx3A, 3B and 4. Transcripts for Stx3A and 3B splice variants, and Stx4 were observed in rat hippocampus (‘Neuron’). As expected, only Stx 3A and not 3B isoforms were detected in control kidney NRK cells (‘NRK’). (B) Western blot analysis revealed that endogenous Stx 1, 3 and 4 proteins were abundant in hippocampal neurons. As expected, Stx 1 expression was absent in kidney NRK cells. Protein from total membrane extracts from cultured hippocampal neurons (lane 1, 50 μg/lane), adult rat hippocampus (Lane 2, 45 μg/lane), and normal rat kidney (NRK) cell line (lane 3, 50 μg/lane), were analyzed by immunoblotting.
(TIF)

S4 Fig Mistargeting of Stx3 does not disrupt somatodendritic polarization of Tfr. Neurons were cotransfected with TfR-GFP in the absence or presence of Stx3, Stx3Δ38, Stx3_AAA_, or Stx4, and surface TfR was detected with anti-GFP antibody. Representative images show that TfR, a somatodendritic cargo, was somatodendritic under all conditions tested (data quantified in Fig 4B). Bar, 20 μm.
(TIF)

S5 Fig Validation of knockdown constructs for shStx3. Cos-1 cells were cotransfected with either myc-tagged rat Stx3A or 3B isoforms together with combinations of three different shRNAs targeting Stx3 (shStx3, #14–16), or with shNontarget shRNA. Two of the shStx3, #14 and #15, encode sequences specific to both Stx3A and Stx3B isoforms, and #16 targets specifically the Stx3A isoform. Cells were harvested and prepared as whole-cell lysates 24 hours after transfection. Representative Western blots of Stx3A and B expression following knockdown from three independent experiments are shown. Western blots were performed with rat anti-myc antibody; GAPDH was used as the loading control. Band intensity showed 78 ± 3% and 62 ± 1% knockdown of Stx3A and 3B, respectively, with shStx3 compared to control after 24 hrs.
(TIF)
