# Supplementary figures and images for: The SNARE Protein Syntaxin 3 Confers Specificity for Polarized Axonal Trafficking in Neurons
Source: PLoS One. 2016 Sep 23;11(9):e0163671. doi: 10.1371/journal.pone.0163671 (PMC5035089; doi:10.1371/journal.pone.0163671)

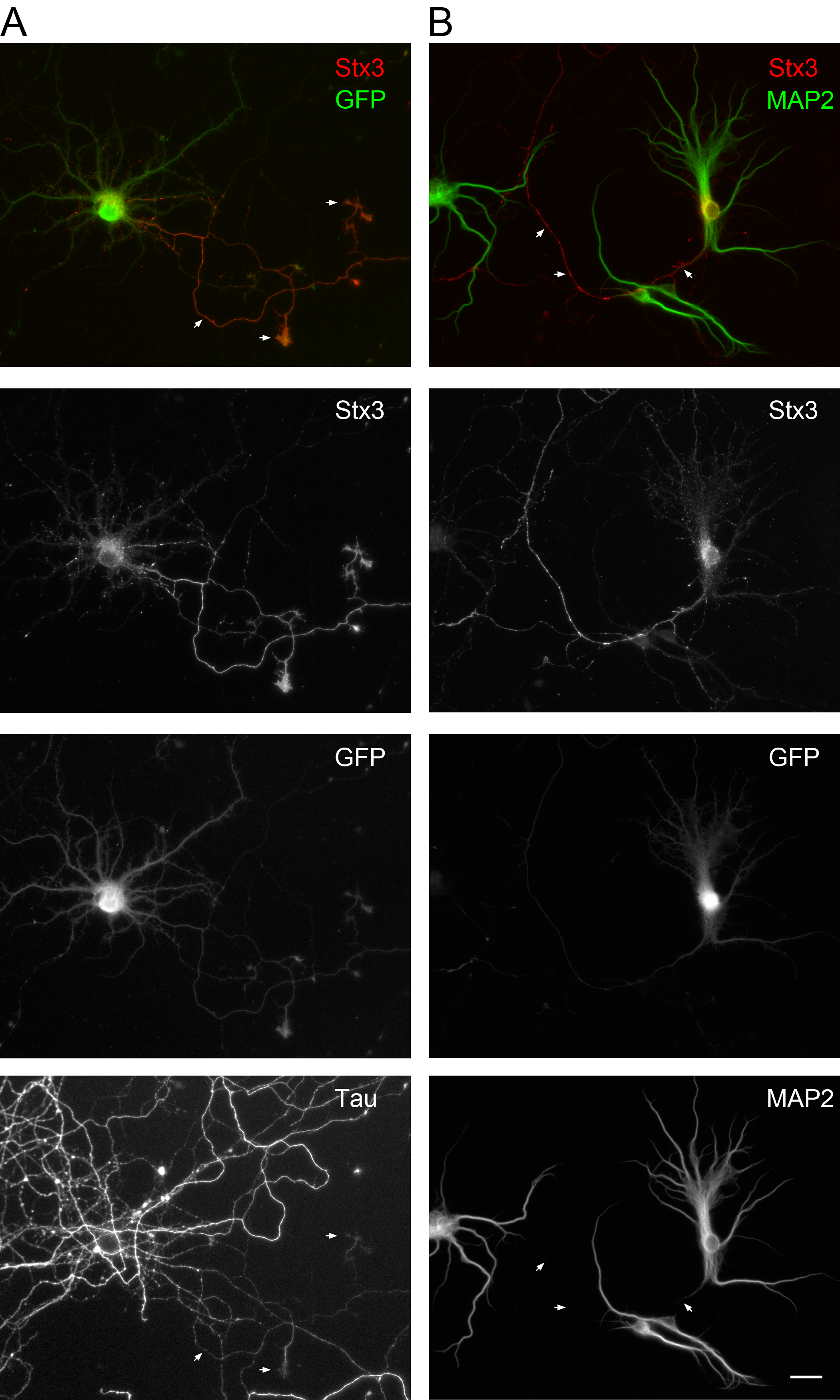

Supplement: S1 Fig — (A) Total Stx3 in neuron infected with adenoviruses encoding Stx3 and GFP (labeled in permeabilized neuron with anti-myc and anti-GFP, respectively) is shown for neuron co-labeled with anti-Tau to identify axons. (B) Total Stx3 expression is similarly shown for a neuron co-labeled with anti-MAP2 to identify dendrites. Arrowheads indicate axon. Bar, 25 μm. (TIF) [file pone.0163671.s001.tif]

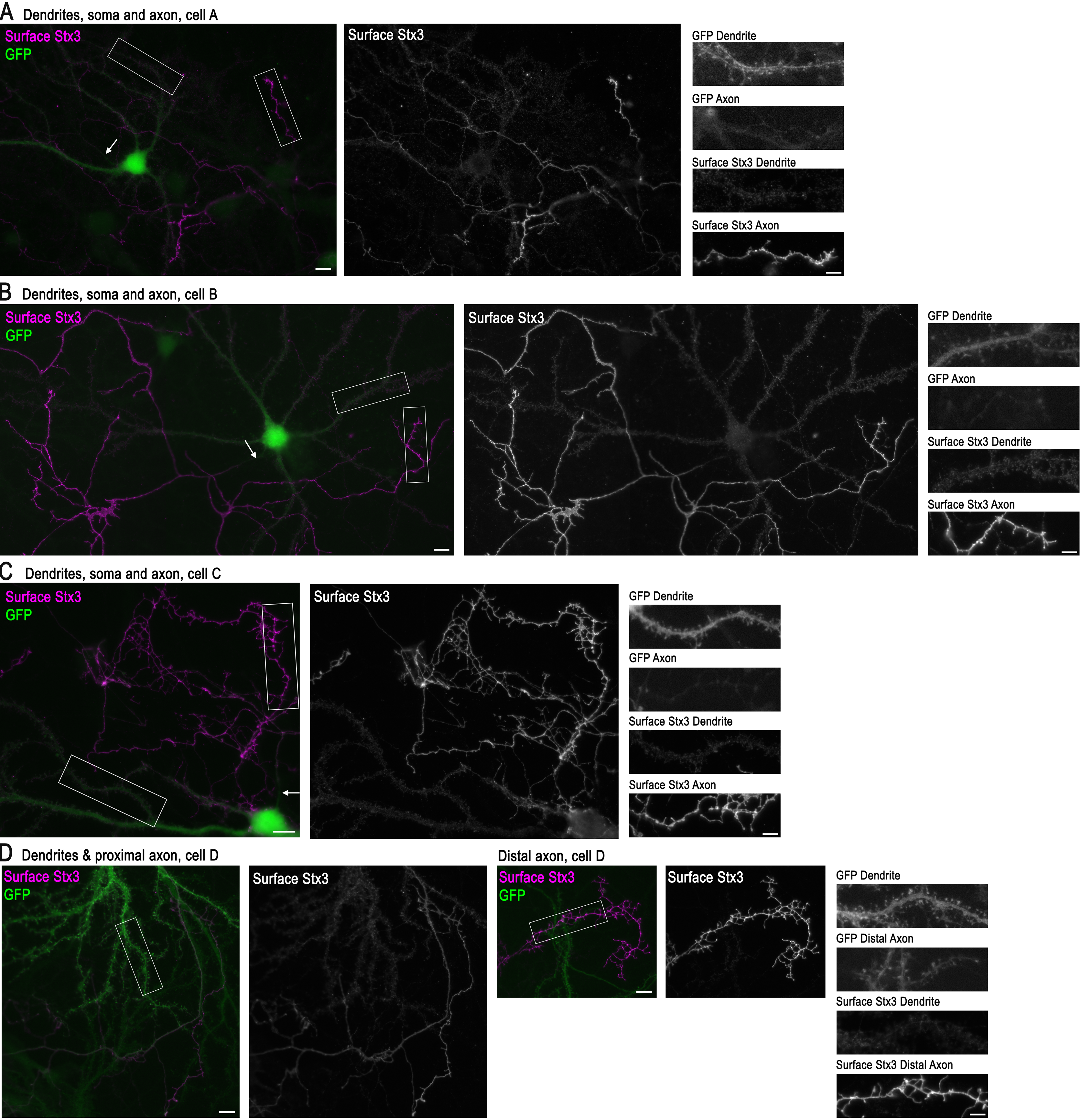

Supplement: S2 Fig — (A-D) Stx3 surface expression is shown for representative neurons (DIV 32) 1 day after infection with adenoviruses encoding Stx3 (surface labeled with anti-myc, then Alexa 647 secondary antibody; magenta) and GFP to highlight cell morphology (labeled with anti-GFP-Alexa 488, green). Boxed regions are enlarged at right to compare intensity and distribution of surface Stx3 and soluble GFP in axons and dendrites. Stx3 surface labeling was bright in axons, particularly at axon tips, and was much lower in dendrites and dendritic spines. Neurons A and C are single images, neuron B is a montage of 2 images with identical exposure times, and neuron D shows proximal and distal regions with identical exposure times. Arrow indicates axon hillock. Bar, 10 μm (images left), 5 μm (insets at right). (TIF) [file pone.0163671.s002.tif]

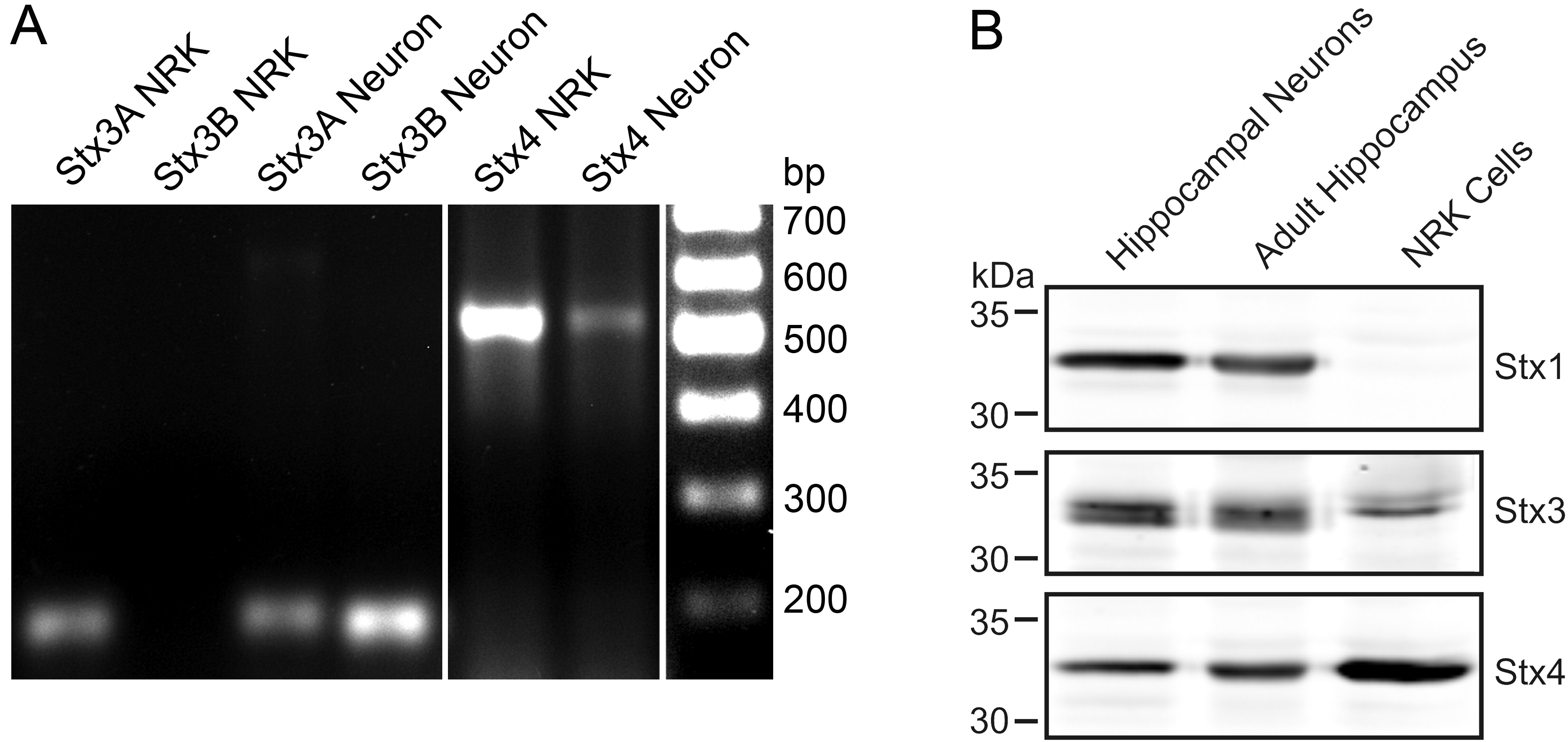

Supplement: S3 Fig — (A) mRNA was amplified by RT-PCR using primer pairs that distinguish Stx3A, 3B and 4. Transcripts for Stx3A and 3B splice variants, and Stx4 were observed in rat hippocampus (‘Neuron’). As expected, only Stx 3A and not 3B isoforms were detected in control kidney NRK cells (‘NRK’). (B) Western blot analysis revealed that endogenous Stx 1, 3 and 4 proteins were abundant in hippocampal neurons. As expected, Stx 1 expression was absent in kidney NRK cells. Protein from total membrane extracts from cultured hippocampal neurons (lane 1, 50 μg/lane), adult rat hippocampus (Lane 2, 45 μg/lane), and normal rat kidney (NRK) cell line (lane 3, 50 μg/lane), were analyzed by immunoblotting. (TIF) [file pone.0163671.s003.tif]

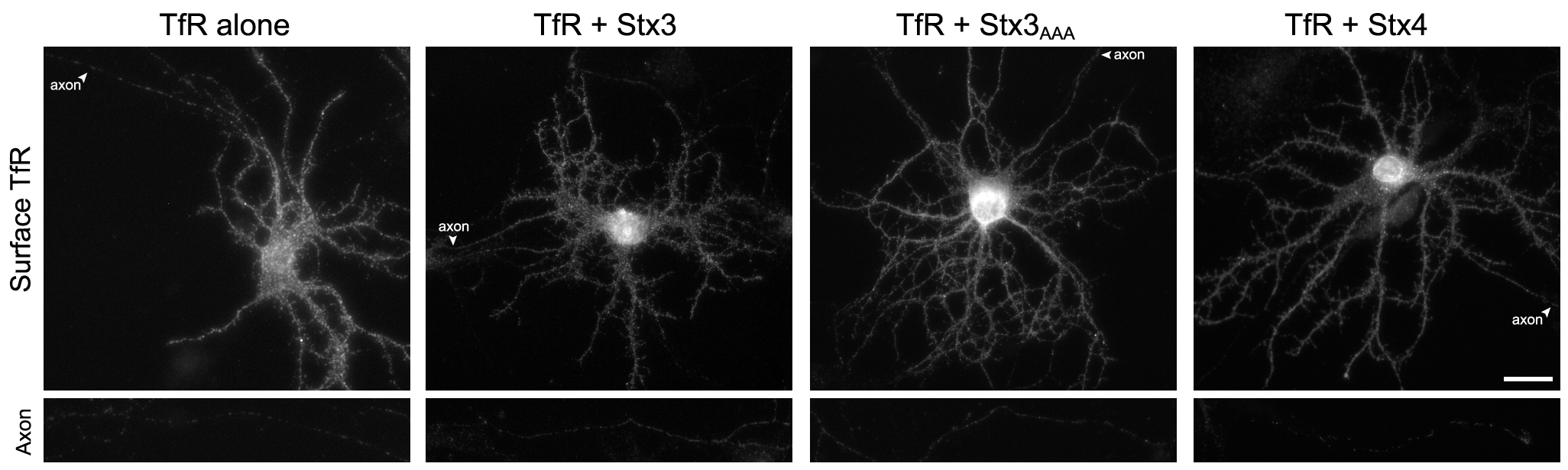

Supplement: S4 Fig — Neurons were cotransfected with TfR-GFP in the absence or presence of Stx3, Stx3Δ38, Stx3AAA, or Stx4, and surface TfR was detected with anti-GFP antibody. Representative images show that TfR, a somatodendritic cargo, was somatodendritic under all conditions tested (data quantified in Fig 4B). Bar, 20 μm. (TIF) [file pone.0163671.s004.tif]

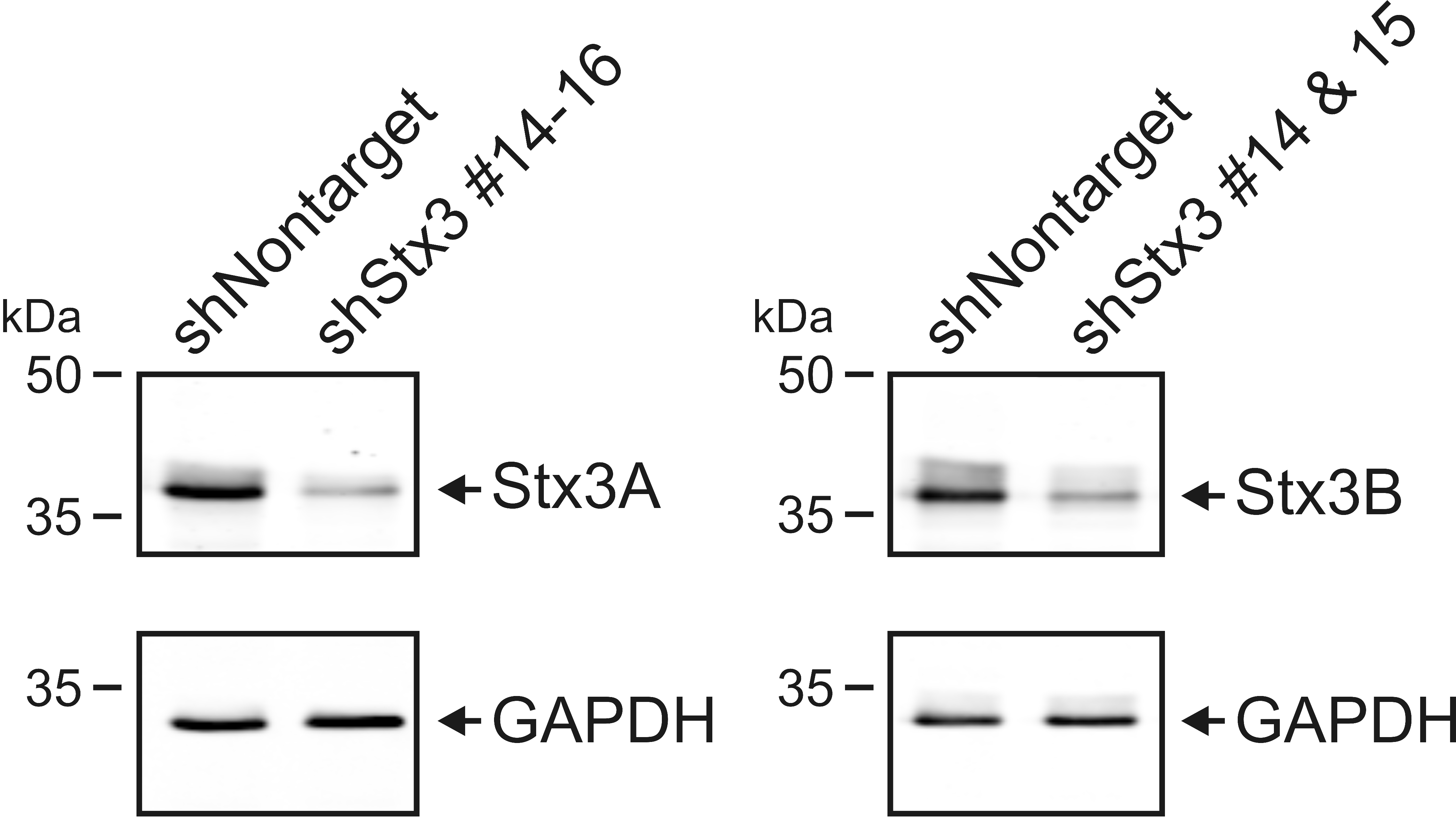

Supplement: S5 Fig — Cos-1 cells were cotransfected with either myc-tagged rat Stx3A or 3B isoforms together with combinations of three different shRNAs targeting Stx3 (shStx3, #14–16), or with shNontarget shRNA. Two of the shStx3, #14 and #15, encode sequences specific to both Stx3A and Stx3B isoforms, and #16 targets specifically the Stx3A isoform. Cells were harvested and prepared as whole-cell lysates 24 hours after transfection. Representative Western blots of Stx3A and B expression following knockdown from three independent experiments are shown. Western blots were performed with rat anti-myc antibody; GAPDH was used as the loading control. Band intensity showed 78 ± 3% and 62 ± 1% knockdown of Stx3A and 3B, respectively, with shStx3 compared to control after 24 hrs. (TIF) [file pone.0163671.s005.tif]
